# Forensic age estimation of adolescents using computed tomography of the clavicles

**DOI:** 10.1007/s00414-024-03272-6

**Published:** 2024-07-03

**Authors:** Leonie Vamberszky, Markus Uhl

**Affiliations:** 1 Medical Center - University of Freiburg, Faculty of Medicine, University of Freiburg, Freiburg, Germany; 2 Department of Radiology, Department of Paediatric Radiology, Medical Center - University of Freiburg, Faculty of Medicine, University of Freiburg, Freiburg, Germany

**Keywords:** Forensic age estimation, Computed tomography, Clavicle, Adolescent

## Abstract

In forensic age estimation, CT imaging of the clavicles is used to determine an age over completed 21 years. If ossification of the medial clavicular epiphysis is complete, young men are assumed to be over 21 years of age. The aim of this study is to check the statistical parameters (specificity, predictive probability) for the characteristic "completed ossification of the medial clavicles". 285 male patients who, for various reasons, received a chest CT at the Medical Center of the University of Freiburg between 1st December 2019 and 6th December 2022 were screened for the study, of whom 203 patients were included in the study. The stage of clavicular ossification was classified as stage 1 – 5 according to Schmeling. While 70 out of 71 patients under 21 years of age were correctly estimated to be under 21 years of age, there was one patient whose ossification on one side was classified as stage 4 and who would therefore have been estimated to be over 21 years of age. If only subjects whose ossification stage was the same on both sides are included, the specificity of the test method is 100% and the positive predictive probability is 100%. If patients for whom only one side is stage 4 are also included, the specificity is 98.6%. Thus, only the complete and symmetrical ossification of both clavicles (stage 4 according to the Schmeling classification) in a standardised thin-layer CT can be classified as a reliable indicator of an age over 21 years in young men. In the case of asymmetric ossification of the medial clavicles (stage 4 is not reached on one side), false positive evaluations and the incorrect assumption of an age over 21 years can occur.

## Introduction

Forensic age estimation is used in adolescents and young adults who are subject to a conviction under juvenile or adult criminal law and whose chronological age cannot be determined with certainty due to a lack of identification documents. According to guidelines from the AGFAD (Study Group on Forensic Age Diagnostics of the German Association of Forensic Medicine), a physical examination is first carried out, in addition to a dental examination to determine the dental status and an X-ray examination of the teeth and left hand. If the ossification of the skeleton of the hand is already complete, CT imaging of the clavicles is carried out [1].

If an expert opinion is required for the forensic age estimate, this may address both the questions of the minimum age and the most likely age of the person in question. If the person states their age, the plausibility of their statement can be checked [2]. According to current clinical practice, a chronological age over 21 years is assumed where there is complete ossification of the clavicles [3].

The decisive criterion for assessing medial clavicular ossification by means of thin-slice CT-imaging for age assessment purposes according to the widely acknowledged typologies of Schmeling et al. [4] and Kellinghaus et al. [5] is the evaluation of epi-/metaphyseal development state. A completely closed epiphyseal plate corresponds to stage 4 (epiphyseal scar visible) or stage 5 (epiphyseal scar no longer visible). In case of side differences between the complements, the side with the more advanced development is applied for outlining a minimum age in forensic practice [6].

If the exceeding of a legally relevant age limit needs to be assessed with the highest standard of proof ("with a probability bordering on certainty"), the minimum age concept is applied. The minimum age is determined by the age minimum of the reference study for the determined characteristic attribute (in this case, the complete ossification of the medial clavicular epiphysis, corresponding to stage 4 or 5); it is the age of the youngest person in the reference population who exhibits the characteristic attribute in question. Application of the minimum age concept ensures that the forensic age of the assessed person is never, under any circumstances, stated as too high, but rather, in practice, is always below the actual age [2].

The aim of this study was to check the correlation between stage-4 or -5 ossification and a chronological age over 21 years. Due to the increasing level of migration worldwide, an increase in the relevance of forensic age estimation is also to be expected. The severity of the consequences of a wrongful conviction under adult criminal law means that an underestimation of age is preferred to an overestimation. Recent studies have reported several cases of individuals under the age of 21 presenting with clavicular ossification stage 4 or 5 [7–12]. However, not all studies reach a high standard, as for example, exclusion criteria might be missing. The body of existing studies on the frequency of overestimation of age when thin-section CT imaging is used in line with guidelines is sparse.

## Materials and methods

The study included 203 male subjects between the ages of 18 and 25 on whom, for various reasons (e.g., trauma, to rule out pulmonary embolism), thoracic CT imaging was performed in the period from 1st December 2019 to 6th December 2022. Subjects with normal anatomical variants of the clavicle (fish-mouth-like depression, bowl-like depression, spanner-like configuration, forked clavicle, multiple ossification centres), previous trauma or surgery of the clavicle, or tumour diseases, or subjects whose chronological age could not be determined with certainty were excluded.

Of 285 screened subjects, 49 subjects were excluded due to tumour disease, 28 subjects due to bilateral normal anatomical variants, 4 subjects due to their age not being able to be determined with certainty, and one subject due to a combination of clavicular trauma on one side and a normal variant on the other. For 33 of the remaining 203 subjects, it was only possible to determine the stage on one side (due to exclusion criteria on the other side, e.g., normal variants or clavicular trauma on one side). Where there was a difference in ossification stage between the two sides, the higher stage of skeletal maturity was counted.

**Fig. 1:**
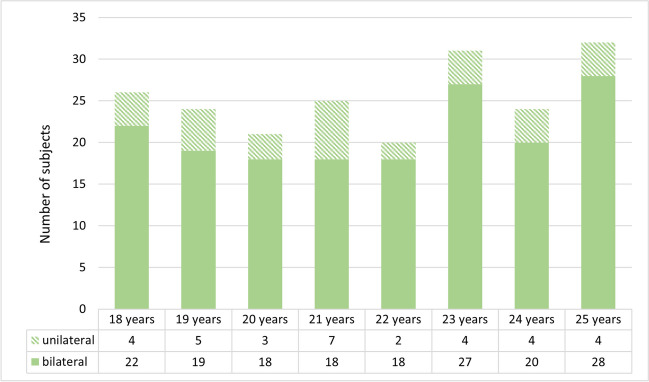
Study population divided into the various age groups. Here, unilateral means that the stage could only be assessed for one of the two clavicles. An exclusion criterion, e.g., a normal variant, is present on the other side. The figure was created using Microsoft Excel

The subjects who could be included in the study were divided into the following age groups (Fig. 1).

The CT scans performed were carried out on a Siemens FLASH CT Scanner (slice thickness 0.75 mm) during the above-mentioned period. Low-dose CTs as well as standard-dose CT images were included in the study, as no significant effect of the dose on the classification into ossification stages has been shown to date [13].

The findings of the CT examinations were checked for exclusion criteria, such as cancers or previous trauma to the clavicles. After calculating the age at the time of imaging, classification into ossification stages was carried out according to Schmeling et al. The age of the subjects was calculated exactly to the day; the length of the year was simplified to 365 days. The classification into an ossification stage was carried out after assessment of the clavicles in two planes (axial and coronal) and based on the guidelines of Wittschieber et al. [14]. The ossification stages were determined per consensus by two readers. While the first reader is a doctoral student, MU has over 30 years of professional experience in the special field of paediatric radiology (including forensic age assessment) and is asked on a regular basis to provide his assessment in legal cases.

## Results

Table 1 shows a breakdown of the different age groups into the five possible ossification stages according to Schmeling et al. In this and the following tables and figures, where there was a difference in ossification stage between the two sides, the side with the higher skeletal maturity was counted.

**Table 1:** Breakdown of subjects by age and ossification stage

Age	Stage 1	Stage 1*	Stage 2	Stage 2*	Stage 3	Stage 3*	Stage 4	Stage 4*	Stage 5	Stage 5*
18 years	5	5	6	9	7	12	0	0	0	0
19 years	2	2	5	6	12	16	0	0	0	0
20 years	0	0	2	2	14	18	0	1	0	0
21 years	0	0	2	3	6	12	7	8	2	2
22 years	0	0	1	1	7	9	1	2	8	8
23 years	0	0	1	1	8	10	10	13	4	7
24 years	0	0	0	0	2	3	5	6	12	15
25 years	0	0	0	0	2	2	6	9	19	21

*Including subjects for whom the stage is only present on one side. Either an exclusion criterion pertains on the opposite side (e.g., normal variant, clavicular trauma), or there is a difference in ossification stage between the sides (in which case, the side that has reached higher skeletal maturity counts)

The data shown above results in the following two-by-two table.

**Fig. 2:**
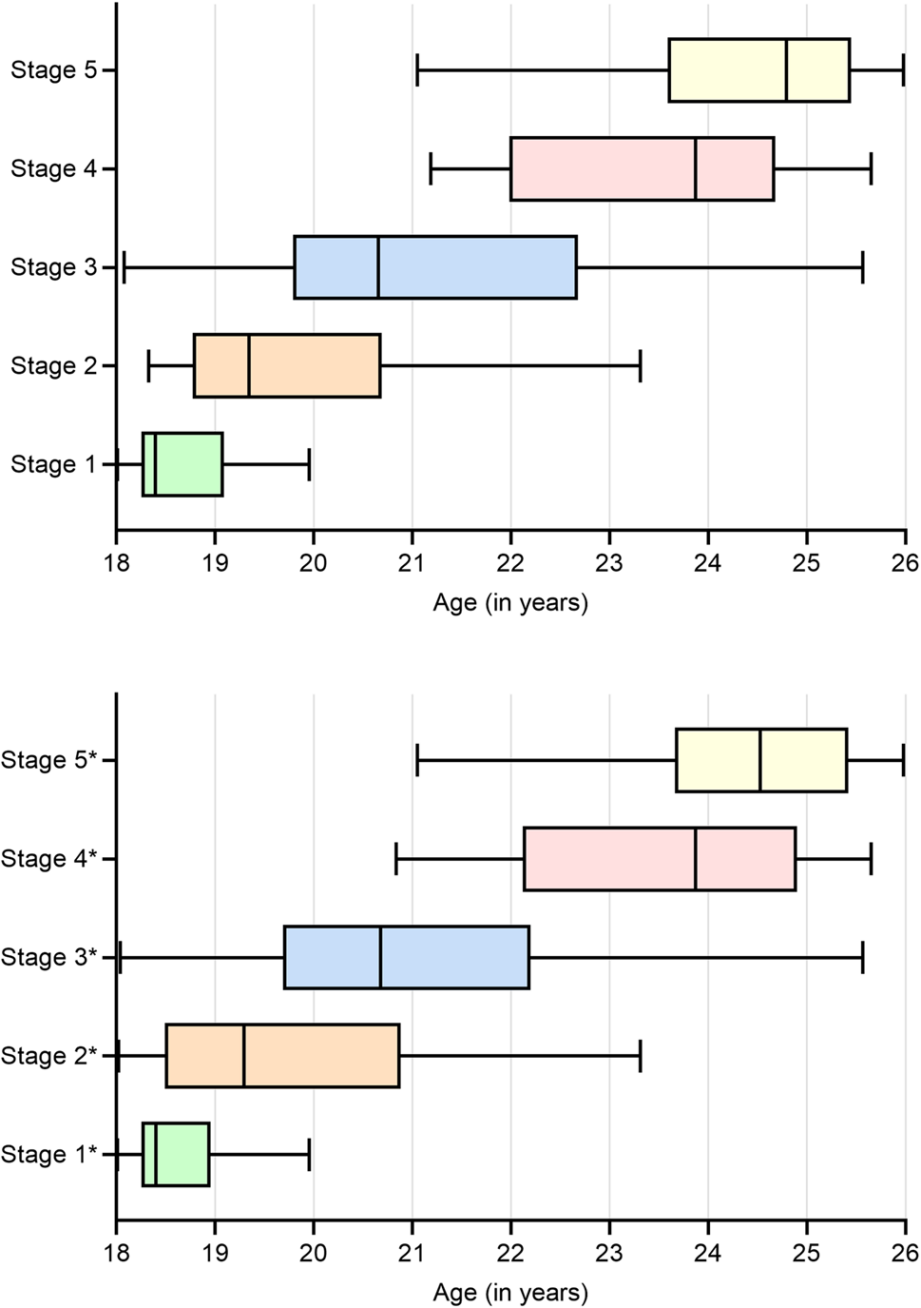
Box plots with correlation of ossification stages with the associated age distribution. The box edges mark the 25% and 75% quartiles, and the centre line marks the median. The lower and upper whiskers indicate the minimum and maximum, respectively. The figure was created using GraphPad Prism. The box plots represent the study population. No conclusions can be drawn on the general populations, as stages may occur in individuals outside of the age range of 18 to 25. *Including subjects for whom the stage is only present on one side. Either an exclusion criterion pertains on the opposite side (e.g., normal variant, clavicular trauma), or there is a difference in ossification stage between the sides (in which case, the side that has reached higher skeletal maturity counts)

Figure 2 shows the data from Table 1 in the form of box plots (minimum, 25% quartile, median, 75% quartile, maximum).

## Conclusion

As shown in Table 2 and Fig. 2, unilateral stage-4 ossification does not allow for the conclusion of an age over 21 years with a probability bordering on certainty. Previous studies, such as the ones conducted by Kellinghaus et al. in 2010 or Wittschieber et al. in 2014, suggested that thin-slice CT imaging as a means of forensic age estimation boasted a specificity of almost 100%, since no subjects under 21 years of age were found to have an ossification stage ≥ 4 [3, 15].

**Table 2:**
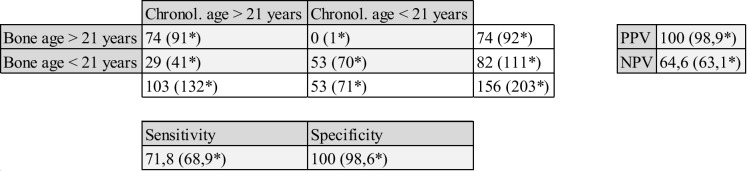
Two-by-two table with calculations for PPV (positive predictive value), NPV (negative predictive value), sensitivity and specificity. For this purpose, a distinction is made between chronological age and bone age (bone age > 21 corresponds to stage 4 or 5 according to Schmeling et al.)

*Including subjects for whom the stage is only present on one side. Either an exclusion criterion pertains on the opposite side (e.g., normal variant, clavicular trauma), or there is a difference in ossification stage between the sides (in which case, the side that has reached higher skeletal maturity counts)

**Fig. 3:**
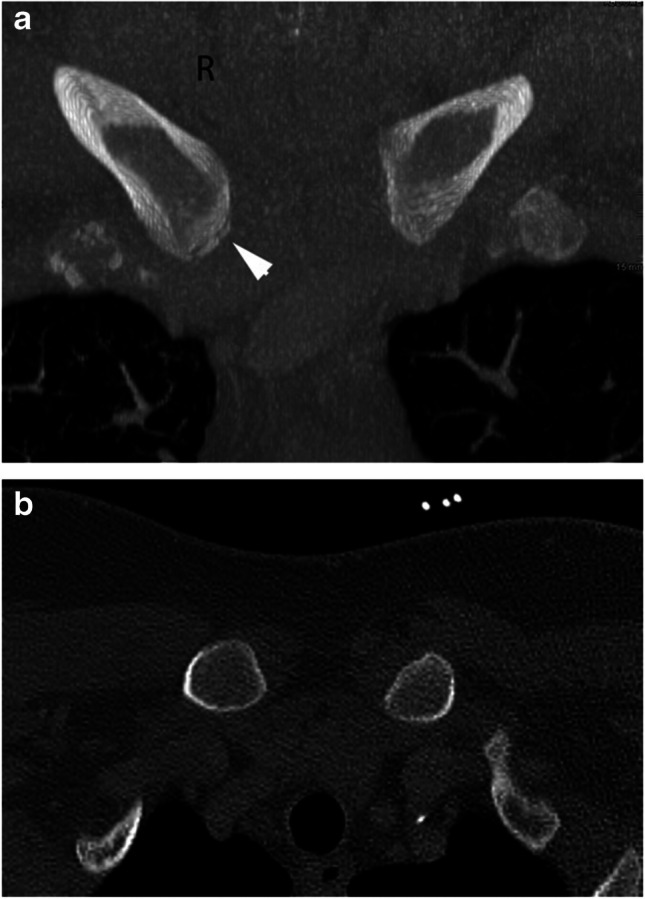
**a/b** Male subject aged 20 years and 10 months. The stages of maturity of the medial clavicles were 3c on the right and 4 on the left (CT MIP projection). He is the only study participant who reached maturity stage 4 before the age of 21. **a** Coronal plane. **b** Axial plane

In this study, only symmetrical, bilateral ossification of stage 4 achieved a specificity and positive predictive probability of 100%. If, on the other hand, only the unilateral attainment of stage 4 is used as a criterion, a specificity of only 98.6% is achieved. In this study population, one subject with a chronological age < 21 years (exact age: 20 years and 10 months) stood out, who exhibited stage-4 ossification on one side and stage 3c on the other side. An expert forensic assessment taking into account only the more advanced side would have diagnosed an age over 21 years. This subject was a heavy labourer (lumberjack) and very muscular, which may explain the premature constitutional skeletal maturity. A possible use of anabolic substances cannot be excluded (Fig. 3a/b).

Furthermore, one subject with the exact age of 21 years and 18 days already exhibited stage-5 ossification, which also suggests complete ossification before the age of 21.

This suggests that the probability of an age over 21 years given the unilateral presence of stage 4 is well over 90%, but not yet at the desired probability of 100%. Overestimation of age is therefore rare, but possible. If stage 4 is present on both sides, or if stage 5 has been reached unilaterally, this probability increases. In previous studies, stage 5 was only found in significantly older men.

## Discussion

Incorrect overestimation of age in criminal proceedings can have serious consequences and should be prevented as far as possible. A specificity well above 99% is to be aimed for, even if the higher specificity is associated with lower sensitivity.

However, in recent years, several studies conducted in multiple different countries have reported cases of individuals below the age of 21 years that reached ossification stage 4 [7–12]. Two studies conducted in Thailand and Japan discussed a possible difference in bone maturation between individuals of Caucasian and Asian descent [11, 12], even though studies by Schmeling et al. and Meijerman et al. had concluded no significant impact of ethnicity on bone maturation [16, 17]. Most of these recent studies present with insufficient exclusion criteria, which is why they are not used as reference studies for forensic age diagnostics [18].

According to the current method, when applying the minimum age concept, the assessed characteristic attribute corresponds to complete ossification of the medial clavicular epiphysis on at least one side [6]. This study indicates that in young men, only attainment of stage 4 or 5 on *both* clavicles can be taken to indicate an age over 21 years with a probability bordering on certainty. If stage 4 or 5 is only reached on one side, age may be overestimated.

Although adjusting the investigated attribute to bilateral attainment of stage 4 would be associated with a reduced sensitivity, with a higher probability of age underestimation, due to the effect of socio-economic status on the time of ossification of the clavicle, which was investigated by Schmeling et al., a high scatter range of age limits with a high probability of age underestimation is generally to be expected [16, 19]. In this study, a higher amount of subjects presented with stage 1 ossification than it would be suspected for this age group. The beforementioned impact of socioeconomic status might have had an impact, as subjects with and without a migration background were included in the study.

A possible limitation of this study is that neither the presence of hormonal diseases nor the use of certain medications were included in the exclusion criteria. It is not suspected that the prevalence of these criteria would be high amongst this age group (especially as the main indication for the CT scans was trauma), but a possible impact on the results cannot be excluded. Particularly with the one subject that stood out, it was described in the radiological findings that he was “very muscular”, which might indicate the use of anabolic substances.

In order to determine the exact percentage of overestimations, studies would have to be carried out with significantly larger study populations. At present, it can be assumed that the specificity of thin-section CT imaging as a test method for age estimation is about 99% or higher, but a specificity of 100% would be desirable.

## Data Availability

The datasets analysed during the current study are not publicly available as the subjects did not explicitly consent to the publication of their CT data sets. A complete CT data set of the subject shown in Fig. 3 is available from the corresponding author on reasonable request.

## References

[1] Arbeitsgemeinschaft für Forensische Altersdiagnostik (AGFAD) (2008) Aktualisierte Empfehlungen für Altersschätzungen bei Lebenden im Strafverfahren. https://www.dgrm.de/fileadmin/PDF/AG_FAD/empfehlungen_strafverfahren.pdf. Accessed 22 August 2023

[2] Schmeling A, Dettmeyer R, Rudolf E, Vieth V, Geserick G (2016) Forensic age estimation—methods, certainty, and the law. Dtsch Arztebl Int 113:44–5. 10.3238/arztebl.2016.0044 26883413 10.3238/arztebl.2016.0044 PMC4760148

[3] Wittschieber D, Schulz R, Vieth V, Küppers M, Bajanowski T, Ramsthaler F, Püschel K, Pfeiffer H, Schmidt S, Schmeling A (2014) The value of sub-stages and thin slices for the assessment of the medial clavicular epiphysis: a prospective multi-center CT study. Forensic Sci Med Pathol 10(2):163–9. 10.1007/s12024-013-9511-x 24277267 10.1007/s12024-013-9511-x

[4] Schmeling A, Schulz R, Reisinger W, Mühler M, Wernicke K-D, Geserick G (2004) Studies on the time frame for ossification of the medial clavicular epiphyseal cartilage in conventional radiography. Int J Legal Med 118(1):5–8. 10.1007/s00414-003-0404-5 14534796 10.1007/s00414-003-0404-5

[5] Kellinghaus M, Schulz R, Vieth V, Schmidt S, Pfeiffer H, Schmeling A (2010) Enhanced possibilities to make statements on the ossification status of the medial clavicular epiphysis using an amplified staging scheme in evaluating thin-slice CT scans. Int J Legal Med 124(4):321–5. 10.1007/s00414-010-0448-2 20354711 10.1007/s00414-010-0448-2

[6] De Tobel J, Ottow C, Widek T, Klasinc I, Mörnstad H, Thevissen PW, Verstraete KL (2020) Dental and skeletal imaging in forensic age estimation: disparities in current approaches and the continuing search for optimization. Semin Musculoskelet Radiol 24:510–522. 10.1055/s-0040-1701495 33036039 10.1055/s-0040-1701495

[7] Ekizoglu O, Hocaoglu E, Inci E, Sayin I, Solmaz D, Bilgili MG, Can IO (2015) Forensic age estimation by the Schmeling method: computed tomography analysis of the medial clavicular epiphysis. Int J Legal Med 129(1):203–10. 10.1007/s00414-014-1121-y 25408292 10.1007/s00414-014-1121-y

[8] Gurses MS, Inanir NT, Gokalp G, Fedakar R, Tobcu E, Ocakoglu G (2016) Evaluation of age estimation in forensic medicine by examination of medial clavicular ossification from thin-slice computed tomography images. Int J Legal Med 130(5):1343–52. 10.1007/s00414-016-1408-2 27352083 10.1007/s00414-016-1408-2

[9] Ufuk F, Agladioglu K, Karabulut N (2016) CT evaluation of medial clavicular epiphysis as a method of bone age determination in adolescents and young adults. Diagn Interv Radiol 22(3):241–6. 10.5152/dir.2016.15355 27015321 10.5152/dir.2016.15355 PMC4859740

[10] Uysal Ramadan S, Gurses MS, Inanir NT, Hacifazlioglu C, Fedakar R, Hizli S (2017) Evaluation of the medial clavicular epiphysis according to the Schmeling and Kellinghaus method in living individuals: A retrospective CT study. Leg Med (Tokyo) 25:16–22. 10.1016/j.legalmed.2016.12.012 28457505 10.1016/j.legalmed.2016.12.012

[11] Pattamapaspong N, Madla C, Mekjaidee K, Namwongprom S (2015) Age estimation of a Thai population based on maturation of the medial clavicular epiphysis using computed tomography. Forensic Sci Int 246:123.e1–5. 10.1016/j.forsciint.2014.10.044 25466155 10.1016/j.forsciint.2014.10.044

[12] Torimitsu S, Makino Y, Saitoh H, Ishii N, Inokuchi G, Motomura A, Chiba F, Yamaguchi R, Hoshioka Y, Urabe S, Iwase H (2019) Age estimation based on maturation of the medial clavicular epiphysis in a Japanese population using multidetector computed tomography. Leg Med (Tokyo) 37:28–32. 10.1016/j.legalmed.2018.12.003 30612021 10.1016/j.legalmed.2018.12.003

[13] Gassenmaier S, Schaefer JF, Nikolaou K, Esser M, Tsiflikas I (2020) Forensic age estimation in living adolescents with CT imaging of the clavicula-impact of low-dose scanning on readers’ confidence. Eur Radiol 30(12):6645–6652. 10.1007/s00330-020-07079-y 32725332 10.1007/s00330-020-07079-y PMC8203536

[14] Wittschieber D, Schulz R, Pfeiffer H, Schmeling A, Schmidt S (2017) Systematic procedure for identifying the five main ossification stages of the medial clavicular epiphysis using computed tomography: a practical proposal for forensic age diagnostics. Int J Legal Med 131(1):217–224. 10.1007/s00414-016-1444-y 27658782 10.1007/s00414-016-1444-y

[15] Kellinghaus M, Schulz R, Vieth V, Schmidt S, Schmeling A (2010) Forensic age estimation in living subjects based on the ossification status of the medial clavicular epiphysis as revealed by thin-slice multidetector computed tomography. Int J Legal Med 124(2):149–54. 10.1007/s00414-009-0398-8 20013127 10.1007/s00414-009-0398-8

[16] Schmeling A, Reisinger W, Loreck D, Vendura K, Markus W, Geserick G (2000) Effects of ethnicity on skeletal maturation: consequences for forensic age estimations. Int J Legal Med. 113(5):253–8. 10.1007/s004149900102 11009058 10.1007/s004149900102

[17] Meijerman L, Maat GJ, Schulz R, Schmeling A (2007) Variables affecting the probability of complete fusion of the medial clavicular epiphysis. Int J Legal Med 121:463–468. 10.1007/s00414-007-0189-z 17909834 10.1007/s00414-007-0189-z PMC2039830

[18] Wittschieber D, Hahnemann ML, Mentzel H (2024) Forensic Diagnostics of the Skeletal Age in the Living – Backgrounds and Methodology. Fortschr Röntgenstr 196:254–261. 10.1055/a-2130-3162 10.1055/a-2130-3162 37699433

[19] Schmeling A, Schulz R, Danner B, Rösing FW (2006) The impact of economic progress and modernization in medicine on the ossification of hand and wrist. Int J Legal Med 120(2):121–6. 10.1007/s00414-005-0007-4 16012824 10.1007/s00414-005-0007-4

